# High-Dose Diosgenin Reduces Bone Loss in Ovariectomized Rats via Attenuation of the RANKL/OPG Ratio

**DOI:** 10.3390/ijms150917130

**Published:** 2014-09-25

**Authors:** Zhiguo Zhang, Changheng Song, Xiaowei Fu, Meijie Liu, Yan Li, Jinghua Pan, Hong Liu, Shaojun Wang, Lihua Xiang, Gary Guishan Xiao, Dahong Ju

**Affiliations:** 1 Institute of Basic Theory, China Academy of Chinese Medical Sciences, Beijing 100700, China; E-Mails: zzgtcm@163.com (Z.Z.); sdsg_sch_86@126.com (C.S.); meimei64571@sina.com (M.L.); lei_ruo@163.com (Y.L.); jh-p@163.com (J.P.); liuhong@163.com (H.L.); wangshaojun@163.com (S.W.); xlh891201@sina.com (L.X.); 2 Clinical Medical College of Chinese and Western Medicine, Shaanxi University of Chinese Medicine, Xi’an 712046, China; E-Mail: xiaoweifu@163.com; 3 Functional Genomics and Proteomics Laboratory, Osteoporosis Research Center, Creighton University Medical Center, Omaha, NE 68131, USA

**Keywords:** diosgenin, bone loss, ovariectomized rats, osteoprotegerin, receptor activator of nuclear factor kappa-B ligand

## Abstract

The aim of this study was to evaluate effect of diosgenin (DG) on rats that had osteoporosis-like features induced by ovariectomy (OVX). Seventy-two six-month-old female Wistar rats were subjected to either ovariectomy (*n* = 60) or Sham operation (SHAM group, *n* = 12). Beginning at one week post-ovariectomy, the OVX rats were treated with vehicle (OVX group, *n* = 12), estradiol valerate (EV group, *n* = 12), or DG at three doses (DG-L, -M, -H group, *n* = 12, respectively). After a 12-week treatment, administration of EV or DG-H inhibited OVX-induced weight gain, and administration of EV or DG-H or DG-M had a significantly uterotrophic effect. Bone mineral density (BMD) and indices of bone histomorphometry of tibia were measured. Levels of protein and mRNA expression of osteoprotegerin (OPG) and receptor activator of nuclear factor kappa-B ligand (RANKL) in tibia were evaluated by immunohistochemistry and *in situ* hybridization. Our results show that DG at a high dose (DG-H) had a significant anti-osteoporotic effect compared to OVX control. DG-H treatment down-regulated expression of RANKL and up-regulated expression of OPG significantly in tibia from OVX rats compared to control, and thus lowered the RANKL/OPG ratio. This suggests that the anti-osteoporotic effect of DG might be associated with modulating the RANKL/OPG ratio and DG had potential to be developed as alternative therapeutic agents of osteoporosis induced by postmenopause.

## 1. Introduction

Osteoporosis, the most common bone remodeling disease, is defined by a low bone mass and a high risk of fractures. Osteoporosis mainly affects postmenopausal women and elderly men. Osteoporosis is caused by an abnormal bone remodeling, *i.e.*, an excess of resorption and less formation, thereby resulting in an increased risk of hip and vertebral fractures [1,2]. The development of bone fragility in postmenopausal women results from rate changes in bone remodeling, leading to alterations of the trabecular bone volume and architecture [3]. In rats, ovariectomy (OVX)-induced bone loss can be treated with estradiol. Because rats and humans share similarities in skeletal responses to estrogen deficiency, the mature OVX rat is considered to be a suitable animal model for studying early postmenopause-induced bone loss [4].

Estrogen, bisphosphonates, parathyroid hormone (PTH), or selective estrogen receptor modulators (SERMs) has been used to prevent the postmenopausal bone loss [5], but many evidences indicate that long-term treatments with those drugs might cause adverse reactions, such as an increased risk of ovarian and endometrial cancer [6,7,8,9], osteonecrosis of the jaws [10], nervous system disorders [11], venous thromboembolism [12], *etc.* Thus, an alternative therapeutic strategy with a proven efficacy should be developed to prevent and treat osteoporosis. Diosgenin (DG), an aglycone of the steroid saponin in *Dioscorea nipponica* or *Rhizoma Dioscoreae* obtained from the hydrolysis of the yam saponins, is the principal raw material in the industrial production of steroid drugs [13]. In a study that assessed the safety of diosgenin-containing yam, it was reported that the expected upper dose limit of diosgenin was 3.5% (*w*/*w*). Although the animals were treated with this dose level of diosgenin, its toxicity or genotoxicity had not been observed [14]. While, some other studies reported that diosgenin treatment showed some side effects, such as stimulating the growth of the mammary gland [15] and the adrenal gland [16]. Recent studies have indicated that DG may protect against bone loss of rats [17,18] and that DG is an estrogen-like compound mediating its effects through estrogen receptor (ER)-dependent pathway [19]. However, the potential therapeutic mechanism of DG on bone loss induced by estrogen deficiency has not been revealed. The interaction between receptor activator of nuclear factor kappa-B ligand (RANKL) and osteoprotegerin (OPG) plays a dominant role in osteoclastogenesis, and the RANKL/OPG ratio is an index of osteoclastogenic stimulation [20]. Given the importance of RANKL/OPG ratio in bone metabolism, this study was to evaluate the effect of the DG on RANKL/OPG ratio in bone tissue of ovariectomized rats.

## 2. Results

### 2.1. Effects of DG on Body Weight and Uterine Weight

As shown in Table 1, the body weight of the OVX group was significantly higher than that of the Sham group. Either estradiol valerate (EV), diosgenin high-dose treatment (DG-H) or diosgenin medium-dose treatment (DG-M) or diosgenin low-dose treatment (DG-L) remarkably inhibited OVX-induced weight gain after 12 weeks of treatment.

OVX caused significant atrophy of uterine tissue compared with the Sham group, indicating the success of the surgical procedure. Compared with the OVX group, administration of EV significantly heightened uterine weight, whereas either DG-H or DG-M for 12 weeks had a mild uterotrophic effect (Table 1). 

**Table 1:** Comparison of body weight and uterine weight among groups after 12-week treatment.

Group	*n*	Body Weight (g)	Uterine Weight (mg)
Sham	12	324 + 18	830 ± 20
OVX	12	439 + 33 ^a^	226 ± 12 ^a^
OVX + EV	12	317 + 22 ^c^	535 ± 18 ^ac^
OVX + DG-L	12	398 + 25 ^ad^	251 ± 11 ^a^
OVX + DG-M	12	381 + 14 ^ac^	282 ± 13 ^ad^
OVX + DG-H	12	363 + 16 ^ac^	353 ± 15 ^ad^

Values are expressed as mean ± standard deviation (SD). ^a^ *p* < 0.01 *vs.* Sham group; ^c^ *p* < 0.01 *vs.* OVX group; ^d^ *p* < 0.05 *vs.* OVX group.

### 2.2. Effects of DG on Bone Mineral Density, Bone Mineral Content and Projected Bone Area

**Figure 1:**
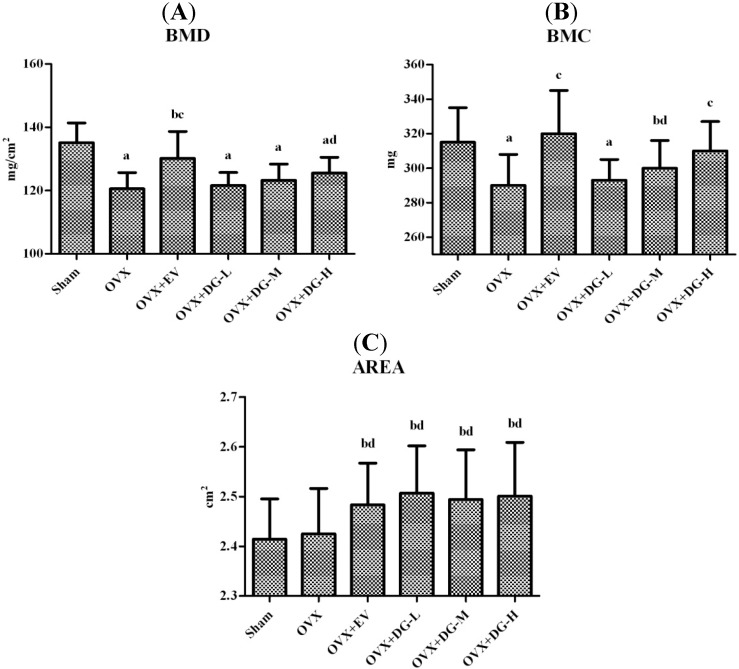
Effects of diosgenin on femoral bone mineral density (BMD), bone mineral content (BMC), and projected bone area (AREA) in ovariectomy (OVX) rats. After 12-weektreatment, femurs were dissected free of soft tissue. The BMD (**A**); BMC (**B**); and AREA (**C**)of the femur were measured by dual-energy X-ray absorptiometry. Results are means± S.D. (*n* = 12 rats/group). a, *p* < 0.01 *vs.* Sham group; b, *p* < 0.05 *vs.* Sham group; c, *p* < 0.01 *vs.* OVX group; d, *p* < 0.05 *vs.* OVX group.

To investigate whether DG has an anti-osteoporotic effect, the bone mineral density (BMD), bone mineral content (BMC), and projected bone area (AREA) of the total femur were measured by dual-energy X-ray absorptiometry (DXA). Figure 1 shows that ovariectomy significantly reduced BMD of the total femur compared to Sham operated animals. EV or DG-H for 12 weeks significantly heightened the BMD of femur compared to the OVX group. 

### 2.3. Effects of DG on Indices of Bone Histomorphometry

**Figure 2:**
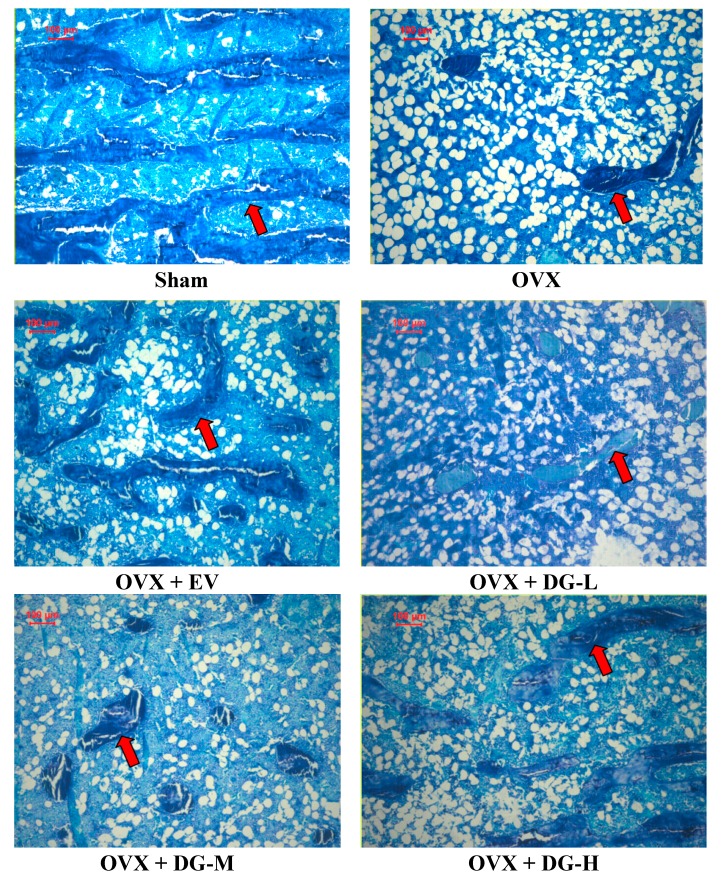
Effects of diosgenin on trabecular bone. Trabecular bone was visualized by using methylene blue staining. The red arrow indicates trabecular bone.

Bone turnover is a life-long process where two counter-balanced processes (bone resorption and bone formation) are involved. Bone turnover can be monitored by measuring histomorphometric indices. To further estimate the effects of DG on bone turnover, we analyzed five bone histomorphometric indices including trabecular bone volume (BV/TV), resorption surface (ES/BS), active forming surface (MS/BS), mineral apposition rate (MAR), osteoid seam width (O.Th) in all rats using methylene blue staining and histomorphometry (Figure 2). Double labels of tetracycline were present in all rats. BV/TV was significantly reduced in rats with estrogen deficiency induced by ovariectomy. Yet, MS/BS, ES/BS, MAR and O.Th were heightened significantly in these OVX rats (Table 2). EV treatment significantly rescued the effects of ovariectomy on histomorphometric indices by increasing BV/TV, and decreasing MS/BS, ES/BS, MAR and O.Th in OVX rats. Compared to OVX rats, only DG-H treatment showed a similar effect to EV treatment on four indices except O.Th (Table 2). 

**Table 2:** Comparison of indices of bone histomorphometry among groups.

Group	*n*	BV/TV (%)	ES/BS (%)	MS/BS (%)	MAR (μm/day)	O.Th (μm)
Sham	12	26.09 ± 2.66	2.35 ± 0.78	4.16 ± 0.72	0.95 ± 0.19	2.20 ± 0.46
OVX	12	15.15 ± 3.55 ^a^	10.35 ± 1.60 ^a^	9.57 ± 1.53 ^a^	2.16 ± 0.27 ^a^	3.55 ± 0.58 ^a^
OVX + EV	12	23.10 ± 2.86 ^c^	2.44 ± 0.94 ^c^	4.54 ± 1.02 ^c^	1.06 ± 0.17 ^c^	2.56 ± 0.33 ^bc^
OVX + DG-L	12	16.08 ± 3.89 ^a^	9.51 ± 2.11 ^a^	9.15 ± 1.43 ^a^	2.06 ± 0.31 ^a^	3.44 ± 0.70 ^a^
OVX + DG-M	12	17.74 ± 4.42 ^a^	8.47 ± 1.86 ^a^	8.75 ± 1.32 ^a^	1.92 ± 0.29 ^a^	3.37 ± 0.55 ^a^
OVX + DG-H	12	19.93 ± 3.71 ^ac^	5.22 ± 0.88 ^ac^	6.01 ± 0.83^ac^	1.67 ± 0.33 ^ad^	3.13 ± 0.71 ^a^

Values are expressed as mean ± standard deviation (SD). ^a^ *p* < 0.01 *vs.* Sham group; ^b^ *p* < 0.05 *vs.* Sham group; ^c^ *p* < 0.01 *vs.* OVX group; ^d^ *p* < 0.05 *vs.* OVX group.

### 2.4. Effects of DG on Expression of RANKL/OPG Ratio

**Figure 3:**
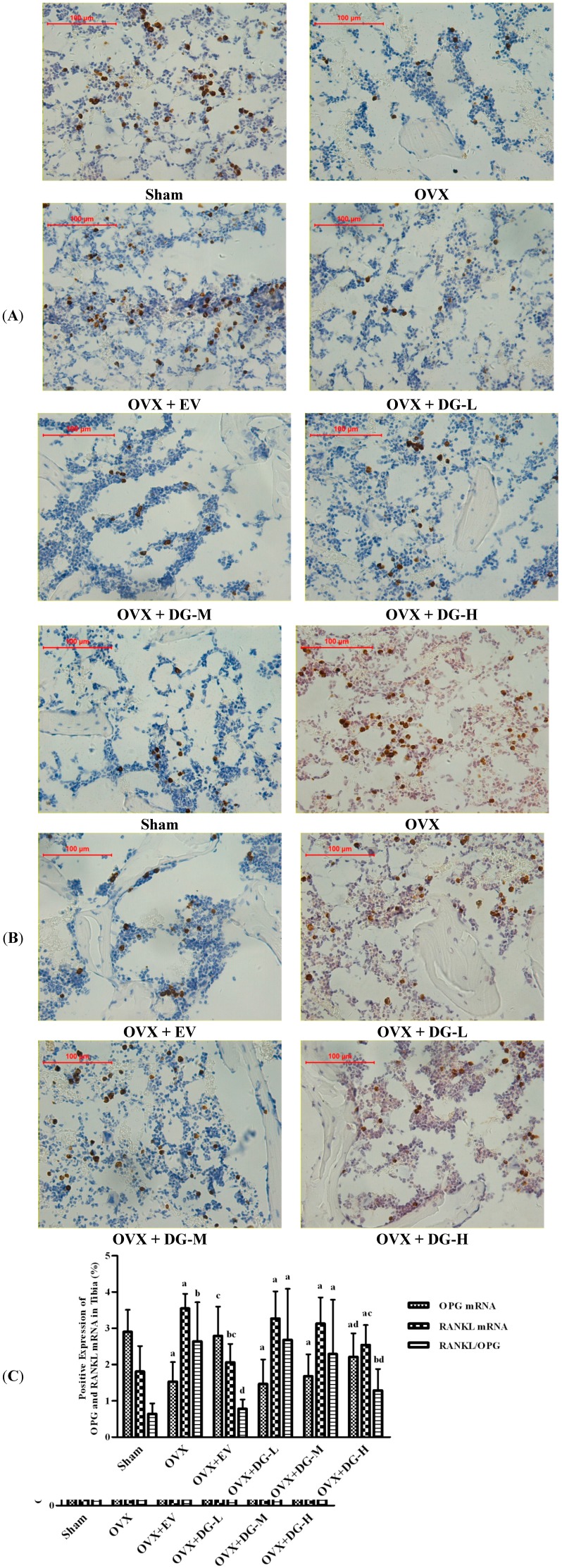
Effects of 12 weeks treatment on expression of OPG and RANKL mRNA in tibiae of rats. Expression level of OPG and RANKL mRNA was estimated by using *in situ* hybridization. (**A**) OPG mRNA expression; (**B**) RANKL mRNA expression, and (**C**) mRNA ratio of RANKL/OPG are shown. In panel (**C**), a, *p* < 0.01 *vs.* Sham group; b, *p* < 0.05 *vs.* Sham group; c, *p* < 0.01 *vs.* OVX group; d, *p* < 0.05 *vs.* OVX group.

**Figure 4:**
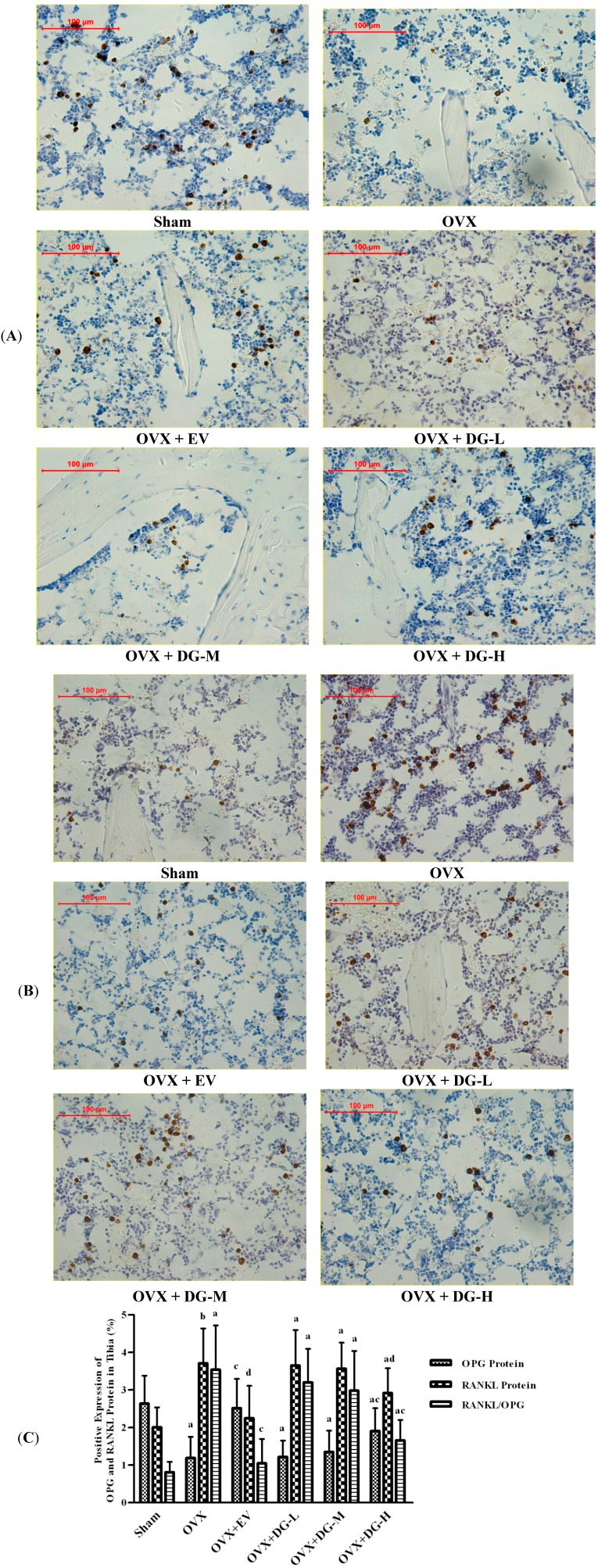
Effects of 12 weeks treatment on expression of OPG and RANKL protein in tibiae of rats. Expression level of OPG and RANKL protein was estimated by immunohistochemical analysis. (**A**) OPG expression; (**B**) RANKL expression; and (**C**) Ratio of RANKL/OPG are shown. In panel (**C**), a, *p* < 0.01 *vs.* Sham group; b, *p* < 0.05 *vs.* Sham group; c, *p* < 0.01 *vs.* OVX group; d, *p* < 0.05 *vs.* OVX group.

To monitor bone turnover in OVX rats treated with DG, we analyzed expression of OPG and RANKL, two important bone turnover factors, using immunohistochemical assessment and *in situ* hybridization (Figure 3 and Figure 4). 

Protein and mRNA expression of OPG were lowered significantly in tibia tissue from OVX rats compared to Sham group. Either EV or DG-H treatment caused overexpression of OPG protein or mRNA in tibia from OVX rats compared to the OVX group, but treatment with DG-L or DG-M had no significant effects on OPG expression (Figure 3A and Figure 4A). Ovariectomy caused a significant rise of protein and mRNA expression of RANKL in tibia compared to Sham group. Compared to the OVX group, protein or mRNA expression of RANKL in tibia from OVX rats reduced significantly after treatment of EV or DG-H, but treatment with DG-L or DG-M had no significant effects on RANKL expression (Figure 3B and Figure 4B). RANKL/OPG expression ratio of mRNA in tibia from six groups was coincident with that of protein. RANKL/OPG ratio was heightened dramatically in tibia tissue from OVX rats compared to Sham group. Compared to OVX group, either EV or DG-H treatment could attenuate RANKL/OPG expression ratio in tibia of OVX rats significantly, but treatment with DG-L and DG-M had no significant effects on RANKL/OPG ratio (Figure 3C and Figure 4C).

## 3. Discussion

Diosgenin, a steroid saponin, is a major bioactive constituent of various edible pulses and roots, well characterized in the seeds of fenugreek (*Trigonella foenum graecum* Linn) as well as in the roots of wild yams (*Dioscorea villosa* Linn) [14,21]. Structurally, diosgenin [(25*R*)-spirost-5-*en*-3*b*-ol] is a spirostanol saponin consisting of a hydrophilic sugar moiety linked to a hydrophobic steroid aglycone. In the pharmaceutical industry, diosgenin is the single main precursor in the manufacture of synthetic steroids [22]. Biological activities of diosgenin have been widely studied. Diosgenin is considered to have therapeutic effects on dyslipidemia/obesity [23], diabetes [24], and vascular calcification [25], *etc*. However, these studies had been done in animals or *in vitro*. 

The RANKL/RANK/OPG system plays a key role in the regulation of bone metabolism [26]. Some studies have reported that diosgenin have an anti-osteoporotic effect on model rats [17,18], but these studies only confirmed the anti-osteoporotic effect of DG preliminarily. Whether or not DG protects against bone loss in model rats via regualation of the RANKL or OPG expression remains unknown. In the present study, we evaluated the effect of DG on expression of RANKL or OPG in osteoporosis rats induced by OVX for the first time. 

In this study, the results show that treatment of DG at different dose for 12 weeks prevented body weight gain and loss of uterine wet weight induced by estrogen deficiency in OVX rats (Table 1). The effect of DG on the body weight and uterus reported in current studies of were conflicting. Some study indicated that DG did not express estrogenic activity and had no substantial effect on body weight gain [27] or uterine atrophy [28] caused by OVX, but other studies had proven the estrogen-like effect of DG on the body weight [29] and uterus [27,30]. Our results supported that DG is a weak estrogen agonist and can retard the body weight gain and uterine atrophy induced by OVX. 

With an ovariectomy, BMD is markedly lowered due to a rise in bone turnover in the OVX rats compared to the Sham rats. DG-H treatment for 12 weeks significantly raised the BMD of the femur compared to untreated OVX rats (Figure 1). BMD is only a measure of areal bone density and does not take into account the histomorphological changes occurring in trabecular bone. There is strong evidence the histomorphological change of cancellous bone plays a significant role in bone strength and determines its biomechanical properties [31]. To observe the histomorphological change of cancellous bone, we had used indices of bone histomorphometry to further explain the change of BMD. 

According to the results of our experiment, the OVX led to significant bone loss in tibia, as shown by BV/TV, an important bone mass index. Furthermore, coincident and significant increasing in indices for assessment of bone resorption, ES/BS, and for assessment of bone formation, MS/BS, MAR and O.Th indicated that mature OVX rat is a good animal model for studying high-turnover osteoporosis such as early postmenopausal osteoporosis [32,33]. Treatment with EV for 12 weeks was able to prevent the bone loss induced by the OVX, which was reflected by the rise in BV/TV, and lower the raised bone-turnover, which was reflected by the reduction in ES/BS, MS/BS, MAR and O.Th significantly. Except O.Th, the effects of DG-H on BV/TV, ES/BS, MS/BS and MAR indicated that DG-H had inhibitory effects on bone-turnover, but. DG-M and -L had no significant inhibitory effects on ES/BS, MS/BS, MAR and O.Th. 

The RANKL/RANK/OPG system plays a key role in the regulation of bone metabolism. RANK is a receptor located on surface osteoclasts (precursor and mature). Ligands of RANK are OPG and RANKL synthesized and secreted primarily by osteoblasts and bone marrow stromal cells [34,35,36]. When RANK is activated by the RANKL, a signaling cascade is initiated, causing osteoclast differentiation is triggered and bone resorption is increased. OPG, which acts as a decoy receptor for RANKL, blocks this interaction and inhibits the activation of osteoclasts. The balance between expressions of RANKL and OPG in osteoblasts and bone marrow stromal cells regulates bone resorption. The importance of this system in bone metabolism is demonstrated by the facts that pharmacologic blockade of RANKL is an effective treatment for osteoporosis [37], that an inherited deficiency of RANK or RANKL causes osteopetrosis, and that loss-of-function OPG mutations cause juvenile Paget’s disease [38,39,40]. 

In this study, we found that OVX down-regulated expression of protein and mRNA of OPG and up-regulated expression of protein and mRNA of RANKL in OVX rat tibia compared with Sham rats, while EV or DG-H treatment modulated this course significantly. The results indicated that one of actions of DG-H inhibiting bone loss lay in modulatory effect on of RANKL/OPG ratio. 

In this study, we found that DG could prevent bone loss in an ovariectomized rat model of osteoporosis, but only DG at a high dose had a significant anti-osteoporotic effect. Our study showed that DG had potential to be developed as alternative therapeutic agent of osteoporosis induced by postmenopause.

## 4. Materials and Methods

### 4.1. Animal Grouping and Treatments

Many studies had shown that 6-month-old female rat ovariectomized bilaterally was a good model for postmenopausal osteoporosis [32,33]. We obtained a total of 72 6-month-old virgin Wistar rats with body weight of 310 ± 20.0 g from the Experimental Animal Center of Academy of Military Medical Sciences (SCXK-(Military) 2002-001, Beijing, China). The Institutional Ethics Committee of China Academy of Chinese Medical Sciences approved the experimental research on the animals. The acclimatized rats were either Sham-operated (SHAM, *n* = 12) or bilaterally ovariectomized (OVX, *n* = 60) using the dorsal approach [41]. The OVX rats were randomly divided into five groups: OVX group (OVX, *n* = 12); estradiol valerate treatment group (OVX + EV, *n* = 12); DG high-dose treatment group (OVX + DG-H, *n* = 12); DG medium-dose treatment group (OVX + DG-M, *n* = 12); DG low-dose treatment group (OVX + DG-L, *n* = 12). The rats in the EV group received estradiol valerate (1mg/tablet, Bayer China Ltd., Shanghai, China), which was dissolved in distilled water, to produce a concentration of 0.1 mg/kg body weight, which was administered daily by oral gavage. According to previous reports [42,43], we use 24 mg/kg body weight/day, 48 mg/kg body weight/day and 96 mg/kg body weight/day as dosages of rats in DG-L, DG-M and DG-H, respectively. The doses of DG are equivalent to 1 time, 2 times and 4 times of the normal human dose of steroidal saponins in clinical prescription for coronary heart disease or myocardial ischemia (1.44 g/60 kg weight/day). The rats in the DG groups were administered DG (Sigma-Aldrich, Saint Louis, MO, USA, purity > 95%, dissolved in distilled water) at three dose daily by oral gavage (1 mL/100 g body weight). The rats in the SHAM and the OVX groups were administered the same volume of distilled water by oral gavage (1 mL/100 g body weight). The treatment started 1 week after surgery for 12 weeks. On the 15th day and the 3rd day before sacrifice, all of the rats received tetracycline (Sigma-Aldrich, Saint Louis, MO, USA) at 30 mg/kg body weight by intraperitoneal injection. The rats in all groups were fed standard rodent chow (Animal Center of the Fourth Military Medical University, Xi’an, China). The body weight of each rat was monitored weekly to assess the effect of the treatments.

### 4.2. Preparation of Specimens

One day following the last treatment, the animals were anesthetized with intraperitoneally injected ketamine at 80 mg/kg body weight, together with xylazine at 12 mg/kg body wieght, and sacrificed by exsanguination. The uterus was dissected out and weighed immediately [44]. Right femurs were dissected free of soft tissue and used for the measurement of bone mineral density (BMD), bone mineral content (BMC), and projected bone area (AREA). Proximal right tibiae were dissected and fixed in 4% paraformaldehyde for 24 h, dehydrated in an ethanol gradient of 80%, 90% and 100% for 2 days at each step, defatted in xylene for 2 days, and embedded in plastic polymer. Undecalcified sections (5 µm) were made by microtome (Reichert-Jung 2040, Leica, Germany) and stained with methylene blue or used for fluorescence morphology observation. Proximal left tibiae were fixed in 4% paraformaldehyde for 24 h and decalcified in 10% EDTA at 4 °C for 3 weeks. After that, decalcified samples were dehydrated in 15% sucrose solution for 10 h. Decalcified sections (5 µm) were made (Reichert-Jung 2040, Leica, Germany) and fixed in acetone and were made for immunohistochemistry and *in situ* hybridization.

### 4.3. DXA Analysis

BMC and AREA of total femurs were measured by dual-energy X-ray absorptiometry utilizing a bone mineral analyzer (DCS-600EX-IIIR; Aloka, Tokyo, Japan), and the small animal software according to a method previously described [45]. BMD equaled BMC divided by AREA. 

### 4.4. Bone Histomorphometric Analysis

Undecalcified tibial sections were used for measuring bone turnover activity. All measurements were performed with the automated upright microscope system (Leica DMB6000B and CTR6000, Leica, Wetzlar, Germany) and image analysis system (Qwin, Leica, Wetzlar, Germany). Five bone histomorphometric indices about bone mass and bone turnover were analyzed including BV/TV (expressed as a percentage), ES/BS (expressed as a percentage), MS/BS [(single-label surface/2 + double-label surface)/bone surface], (expressed as a percentage), MAR (micrometer/day) and O.Th (micrometer). All histomorphometric parameter measurements were performed at the metaphyseal region, which was located 1–4 mm from the lowest point of the growth plate and 1 mm from the lateral cortex, excluding the endocortical region [46]. The selected region is known as the secondary spongiosa area, which is rich in trabecular bone. The selected region is squared and the area of the squared region is about 9 mm^2^ (3 mm × 3 mm). In this region, we move the stage and randomly captured five images (without overlapped) with 400× magnifications. All histomorphometric indices were reported according to the standardized nomenclature recommended by the American Society of Bone and Mineral Research [47]. All 12 rats in each group were evaluated and all animal data were obtained by blind measurements.

### 4.5. Immunohistochemical Analysis

The decalcified sections were mounted on glass slides and used for immunohistochemical assessment. Protein expression of OPG and RANKL in tissue sections was detected by using anti-rat antibodies of either RANKL or OPG (Santa Cruz, Santa Cruz, CA, USA). The sections were rinsed in TBS and immersed in 0.3% hydrogen peroxide for 5 min. The slides were then incubated with specific antibodies for 1 h at 37 °C, then rinsed with TBS three times for 3 min. Sections were then incubated with the appropriated unbiotinylated secondary antibody (Zhongshan Goldenbridge, Beijing, China) for 30 min at 37 °C. Slides were then treated with a solution containing DAB (1,4-dideoxy-1,4-imino-d-arabinitol-diaminobenzidine; Sigma, Saint Louis, MO, USA) incubated for 3 min, and rinsed by running water. After that, it was counterstained with Harris hematoxylin and then sealed. As negative control, nonimmune goat serum was used instead of the primary antibody.

All measurements were performed with the automated upright microscope system (Leica DMB6000B and CTR6000) and image analysis system (Qwin, Leica, Wetzlar, Germany). All histomorphometric parameter measurements were performed at the metaphyseal region, which was located 1–4 mm from the lowest point of the growth plate and 1 mm from the lateral cortex, excluding the endocortical region [46]. The selected area is known as the secondary spongiosa area, which is rich in trabecular bone. Five images within measured region were randomly captured with 400× magnifications. There were no overlapping regions between images. The positive immunostained area in a total area of microscopic field was calculated. The positive density of was percentage of positive labeled area under high power field.

### 4.6. Preparation of Riboprobe and in Situ Hybridization Analysis

Total RNAs were extracted from Wistar rats bone and spleen, and BALB/c mouse bone using the SV total RNA isolation system (Promega, Madison, WI, USA) and then a reverse-transcription polymerase chain reaction (RT–PCR) was performed. The primer sets were designed to amplify OPG and RANKL, respectively (Table 3). All selected regions in the mouse contain completely homologous sequences with those in the rats. The suitably digested PCR products were ligated into the pGEM-3Z Vector (Promega) to synthesize both anti-sense and sense probes. The ligated plasmids were then transformed into *Escherichia coli* DH5α competent cells and positive clones were selected. The linearized plasmids were transcribed with T7 or SP6 polymerase and labeled with digoxigenin-UTP using the DIG RNA labeling kit (Roche Diagnostics, Mannheim, Germany). All of the inserted DNA fragments were precisely confirmed by dideoxy sequencing.

The decalcified sections were immersed in solution of 30% hydrogen dioxide and methanol for 30 min, and then incubated with pepsin diluted by 3% citric acid at 37 °C. After that, the sections were postfixed in 1% paraformaldehyde for 10 min. Sections were then incubated with the DIG-labeled antisense cRNA probes at 38–42 °C overnight in a humidified chamber. Posthybridization washes were preceded by multiple washes in 4× SSC at room temperature. Slides were incubated in a blocking reagent for 30 min at 37 °C, and then incubated with a biotinylated anti-digoxin antibody for 60 min, SABC for 20 min and the biotinylated peroxydase for 20 min at 37 °C in turn. Staining was performed with DAB (Sigma, Saint Louis, MO, USA). Finally, sections were covered with glycerol-gelatin and coverslips. 

**Table 3:** The primer sequence used in the experiments (BORSTER, Wuhan, China).

Target	Genbank ID	Primer Sequence (5'-3')
OPG	NM_012870	Forward: 5'-TGGACAACCCAGGAAACCTTTCCTCCAAAA-3'
		Reverse: 5'-TTTGCCTGGGACCAAAGTGAATGCAGAGAG-3'
		Probe: 5'-AGAAATGATAGGGAATCAGGTTCAATCAGT-3'
RANKL	NM_057149	Forward: 5'-GCCAGCCGAGACTACGGCAAGTACCTGCGC-3'
		Reverse: 5'-GGCCAGGTGGTCTGCAGCATCGCTCTGTTC-3'
		Probe: 5'-TTTATAGAATCCTGAGACTCCATGAAAACG-3'

All measurements were performed with the automated upright microscope system (Leica DMB6000B and CTR6000) and image analysis system (Qwin, Leica, Wetzlar, Germany). All histomorphometric parameter measurements were performed at the metaphyseal region, which was located 1–4 mm from the lowest point of the growth plate and 1 mm from the lateral cortex, excluding the endocortical region [46]. The selected area is known as the secondary spongiosa area, which is rich in trabecular bone. Five images within measured region were randomly captured with 400× magnifications. There were no overlapping regions between images. The positive immunostained area in a total area of microscopic field was calculated. The positive density of was percentage of positive labeled area under high power field.

### 4.7. Statistical Analysis

All values were expressed as mean ± standard deviations. All analyses were carried out using the SPSS 13.0 (SPSS Inc., Chicago, IL, USA). The difference between the groups regarding the evaluated parameters was tested by using ANOVA test followed by least significant difference (LSD) test. The data of all groups passed the Kolmogorov-Smirnov test of normality. Significance was accepted when *p* < 0.05. There existed the possibility of type 1 error due to multiple comparisons. 

## 5. Conclusions

This study demonstrated that DG has potential protective effects on ovariectomy-induced osteoporosis in rats in a dose-dependent manner. However, only DG at a high dose had a significant anti-osteoporotic effect. DG at a high dose had an inhibitory effect on both bone formation and resorption synchronously and this inhibitory effect of DG on OVX-induced bone loss is associated with regulation of RANKL/OPG expression ratio. Our study provides evidence that DG may have potential use in the complementary and alternative treatment of postmenopausal osteoporosis.
